# Comparison of whole blood on filter strips with serum for avian influenza virus antibody detection in wild birds

**DOI:** 10.1093/conphys/coaf033

**Published:** 2025-06-09

**Authors:** Jolene A Giacinti, Ishraq Rahman, Jordan Wight, Hannah Lewis, Liam U Taylor, Jennifer F Provencher, Robert Ronconi, Yohannes Berhane, Wanhong Xu, Dmytro Zhmendak, Sailendra N Sarma, Christopher M Sharp, Joshua T Cunningham, April Hedd, Johanna-Lisa Bosch, Gregory J Robertson, Kathryn E Hargan, Andrew S Lang

**Affiliations:** Science and Technology Branch, Ecotoxicology and Wildlife Health Division, Environment and Climate Change Canada, Government of Canada, 1125 Colonel By Drive, Ottawa, ON, K1S 5B6, Canada; Department of Biology, Memorial University of Newfoundland, 45 Arctic Avenue, St. John’s, NL, A1C 5S7, Canada; Department of Biology, Memorial University of Newfoundland, 45 Arctic Avenue, St. John’s, NL, A1C 5S7, Canada; Ontario Region Wildlife and Habitat Assessment Section, Canadian Wildlife Service, Environment and Climate Change Canada, Government of Canada, 335 River Road, Ottawa, ON, K1V 1C7, Canada; Biology Department, Bowdoin College, 255 Maine Street, Brunswick, ME, 04011, USA; Science and Technology Branch, Ecotoxicology and Wildlife Health Division, Environment and Climate Change Canada, Government of Canada, 1125 Colonel By Drive, Ottawa, ON, K1S 5B6, Canada; Atlantic Region Wildlife and Habitat Assessment Section, Canadian Wildlife Service, Environment and Climate Change Canada, Government of Canada, 17 Waterfowl Lane, Sackville, NB, E4L 1G6, Canada; National Centre for Foreign Animal Disease, Canadian Food Inspection Agency, Government of Canada, 1015 Arlington Street, Winnipeg, MB, R3E 3M4, Canada; National Centre for Foreign Animal Disease, Canadian Food Inspection Agency, Government of Canada, 1015 Arlington Street, Winnipeg, MB, R3E 3M4, Canada; National Centre for Foreign Animal Disease, Canadian Food Inspection Agency, Government of Canada, 1015 Arlington Street, Winnipeg, MB, R3E 3M4, Canada; Science and Technology Branch, Ecotoxicology and Wildlife Health Division, Environment and Climate Change Canada, Government of Canada, 1125 Colonel By Drive, Ottawa, ON, K1S 5B6, Canada; Ontario Region Wildlife and Habitat Assessment Section, Canadian Wildlife Service, Environment and Climate Change Canada, Government of Canada, 335 River Road, Ottawa, ON, K1V 1C7, Canada; Science and Technology Branch, Wildlife Research Division, Environment and Climate Change Canada, Government of Canada, 6 Bruce St, Mount Pearl, NL, A1N 4T3, Canada; Science and Technology Branch, Wildlife Research Division, Environment and Climate Change Canada, Government of Canada, 6 Bruce St, Mount Pearl, NL, A1N 4T3, Canada; Science and Technology Branch, Wildlife Research Division, Environment and Climate Change Canada, Government of Canada, 6 Bruce St, Mount Pearl, NL, A1N 4T3, Canada; Science and Technology Branch, Wildlife Research Division, Environment and Climate Change Canada, Government of Canada, 6 Bruce St, Mount Pearl, NL, A1N 4T3, Canada; Department of Biology, Memorial University of Newfoundland, 45 Arctic Avenue, St. John’s, NL, A1C 5S7, Canada; Department of Biology, Memorial University of Newfoundland, 45 Arctic Avenue, St. John’s, NL, A1C 5S7, Canada

**Keywords:** Antibody detection, avian influenza virus, avian serosurveillance, ELISA, filter strip, influenza A virus (IAV), serology, threshold optimization, wild birds

## Abstract

Serological surveillance enhances our understanding of influenza A virus (IAV) exposure and dynamics in wild bird populations. Traditional serum-based testing, while effective, poses logistical challenges for large-scale surveillance, particularly in remote regions, for small-bodied species or in scenarios such as hunter-harvested samples where serum collection can be impractical. This study evaluates the use of whole blood collected on high-quality cellulose filter strips as an alternative to serum for detecting antibodies against IAV nucleoprotein (NP) and hemagglutinin (HA) H5 and H7 targets. We tested paired serum and whole blood on filter strips collected from wild birds using the commercially available IDEXX AI MultiS Screen Ab test and in-house competitive enzyme-linked immunosorbent assays (ELISAs) developed at the National Centre for Foreign Animal Disease (NCFAD) of the Canadian Food Inspection Agency. Strong correlations (*ρ* = 0.77) were observed between serum and whole blood on filter strips for NP detection with the IDEXX ELISA, while moderate correlations were noted for NCFAD’s NP (*ρ* = 0.58) and H5 (*ρ* = 0.65) assays. Correlation between serum and whole blood on filter strips for NCFAD’s H7 assay was poor, although interpretation is limited due to the small sample size of H7 positives. Threshold optimization using the Youden index improved diagnostic performance, with optimized cutoffs identified for NP (sample-to-negative < 0.7708 for IDEXX and percentage inhibition [PI] > 39.56 for NCFAD) and H5 (PI > 20.37). Storage conditions impacted performance, with frozen whole blood on filter strips achieving higher sensitivity compared to those stored at room temperature. These findings support the use of filter strips to collect whole blood as an informative alternative for IAV serological surveillance in wild birds when serum is unavailable, provided optimal storage conditions and threshold adjustments are implemented, although serum remains the superior sample type.

## Introduction

Influenza A viruses (IAVs) are a significant One Health concern, affecting domestic animals, wildlife and humans (Giacinti et al., 2024; Koopmans *et al.,* 2024). Highly pathogenic avian influenza viruses in the H5 goose/Guangdong (Gs/GD) lineage have caused numerous outbreaks in wild birds over the past two decades (Sims *et al.,* 2005; Olsen *et al.,* 2006; Lee *et al.,* 2015). More recently, H5 outbreaks originating in Eurasia (Lycett *et al.*, 2020; Zecchin *et al.,* 2021; Banyard *et al.,* 2022) in 2020 have spread to North America (Alkie *et al.,* 2022; Caliendo *et al.,* 2022), South America (Bruno *et al.,* 2023) and Antarctica Banyard *et al.,* 2024, resulting in widespread morbidity and mortality among wild bird species (Avery-Gomm *et al.,* 2024; Giacinti *et al.*, 2024; Lane *et al.,* 2024; McPhail *et al.,* 2024). These mass die-offs have raised concerns about population-level impacts due to circulation in wild bird populations (Avery-Gomm *et al.,* 2024; Nalepa *et al.,* 2024).

Monitoring viral transmission and mortality is essential for understanding immediate population effects, but paired serological surveillance offers a broader window for inference to better understand viral exposure and survival, which are critical factors for assessing susceptibility and future risk for wild bird populations (Wight *et al.,* 2024). Given the mass die-offs linked to H5N1 in Canada in 2022, understanding the extent of exposure and survival at both individual and population levels can provide important information for decision makers and conducting risk assessments (Nalepa *et al.,* 2024). Although there are commercially validated kits for anti-IAV antibody detection, laboratory-specific methods have also been developed, for example, by the National Centre for Foreign Animal Disease (NCFAD) of the Canadian Food Inspection Agency (Yang *et al.,* 2008; Hochman *et al.,* 2023), and standardization across laboratories is essential for effective large-scale surveillance.

Although serum samples are widely recognized as the gold standard for assessing antibodies against IAV in birds, their collection poses significant logistical challenges, particularly for small-bodied or hunter-harvested species and in remote or resource-limited settings. In Canada, harvested species are among the prioritized groups for IAV testing (Nalepa *et al.,* 2024), and hunter–harvester partnerships can provide broad geographic and species coverage for IAV testing. However, submissions often consist of frozen carcasses where whole blood for serum separation is not feasible. Additionally, for smaller-bodied species, including some listed under Canada’s Species at Risk Act (SARA) (Species at Risk Act, 2022), limited blood volumes further complicate serum collection, and require skilled venipuncturists due to the small size of veins.

Even when trained personnel are available, obtaining serum from whole blood requires centrifugation, ideally within 24 hours of collection, which is often difficult in field conditions that frequently lack necessary equipment or storage facilities. While small battery powered centrifuges and serum separator tubes may help, these challenges are exacerbated during extended fieldwork without access to laboratory resources, making it difficult to prevent hemolysis and ensure sample integrity (Spackman, 2014). As a result, alternative blood collection methods that address these logistical limitations can facilitate large-scale and remote surveillance programmes.

**Figure 1:**
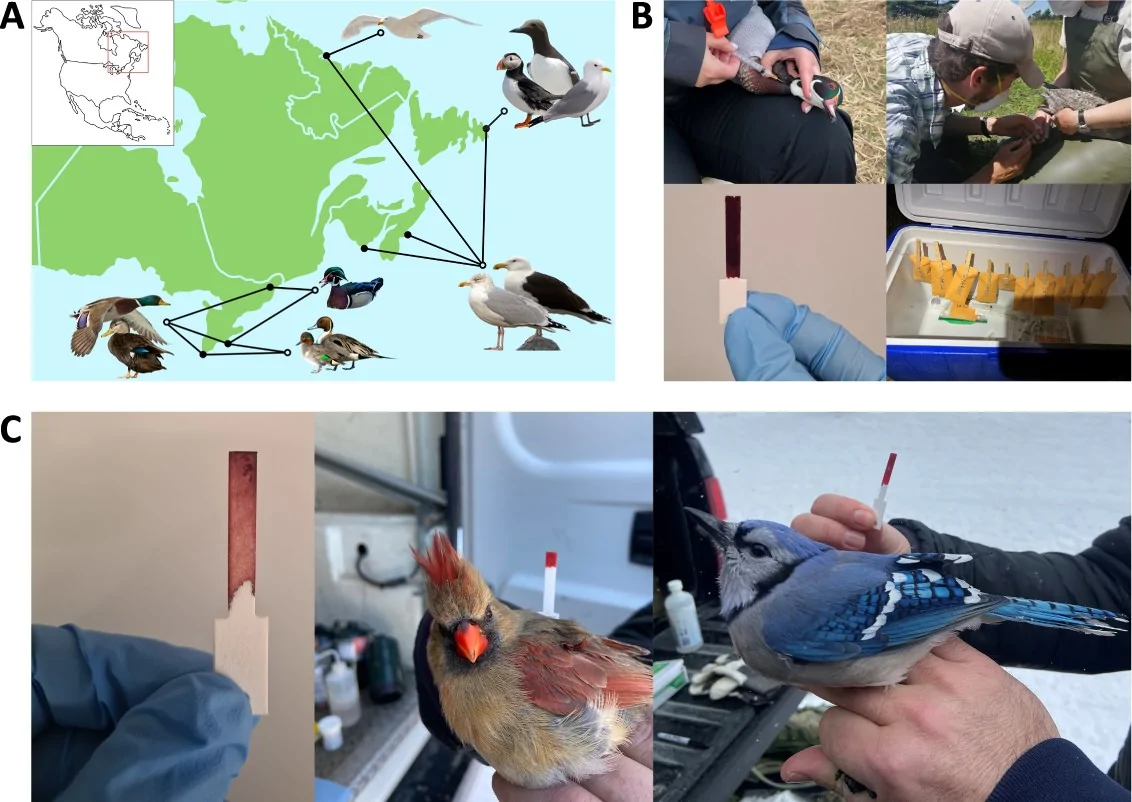
(**a**) Map of sampling locations and species included in the study. Sampling sites span various regions in Canada and represent diverse wild bird species from three taxonomic families: Anatidae, Alcidae and Laridae (photo credit: P. Reeves/J. Whittingham/C. Birdman/N. Bowman/L. Gin/K. Bock/K. Boks via Canva.com); (**b**) field photos illustrating key steps in sample collection using filter strips, including venipuncture of wild birds via the jugular (photo credit: R. Wood) and medial metatarsal vein (photo credit: I. Kyle; brachial not shown), saturation of filter strips with whole blood (photo credit: I. Rahman) and a field drying method using tented paper coin envelopes to ensure complete drying of the strips prior to storage (photo credit: J. Giacinti). (**c**) These images do not depict species or samples used in the analyses but illustrate practical use cases for filter strip and where standardized protocols for adjusting elution volumes may be necessary. The first image shows a filter strip from a hunter-harvested bird that was not fully saturated (photo credit: I. Rahman), while the following images depict small-bodied land birds where collecting sufficient blood for full saturation was impractical or inadvisable (photo credit: R. Wood/J. Giacinti).

Whole blood collected on filter strips provides a relatively simpler and more accessible alternative for obtaining samples from some species and settings. Developed initially for diagnosing toxoplasmosis (Nobuto, 1967), these high-purity cellulose filter strips absorb a uniform amount of blood and are easy to use. Filter strips have been employed in various wildlife seroprevalence studies, including for parvovirus and canine distemper in coyotes, *Brucella* in caribou and Newcastle disease virus in birds (Beard and Brugh, 1977; Avakian and Dick, 1985; Curry *et al.,* 2011; Kamps *et al.,* 2015). Despite their advantages, the efficacy of whole blood samples collected on filter strips for the detection of antibodies varies depending on the antibody of interest, the collection method and storage conditions, as well as the time between sample collection and testing (Kunkel *et al.,* 2023). To date, only one study has validated the use of blood samples collected on filter strips for detecting antibodies against IAVs in waterfowl (Dusek *et al.,* 2011). However, Dusek *et al*. (2011) carried out their study in a controlled laboratory setting, with optimal sample collection, and only screened for anti-nucleoprotein (NP) IAV antibodies. Further validation under field conditions and across antibody targets is needed to support their broader application.

This study aimed to compare the performance of filter strips saturated with whole blood against serum for detecting anti-NP, anti-H5 and anti-H7 antibodies using IDEXX and NCFAD enzyme-linked immunosorbent assays (ELISAs) in wild bird species. We also assessed the need for threshold optimization—adjusting the cutoff values used to distinguish between positive and negative test results to maximize diagnostic sensitivity and specificity—to improve diagnostic performance of whole blood on filter strips for detecting these antibodies. Finally, we evaluated the impact of storage conditions (room temperature versus frozen at −20°C) on the performance of filter strip samples in detecting anti-NP and anti-H5 antibodies. We predicted that whole blood on filter strips would have lower sensitivity and specificity compared to serum for detecting anti-IAV antibodies but did not expect performance of tests directed at different antibody targets (i.e. NP, H5, H7) to vary. Given that previous studies have shown storage conditions can influence test performance, we anticipated that freezing whole blood on filter strips would enhance sensitivity and specificity.

## Materials and Methods

### Field sampling

Live wild birds trapped in 2023 and 2024 as part of ongoing banding or research programmes contributed to Canada’s Interagency Surveillance Program for Avian Influenza Viruses in Wildlife (Giacinti *et al.*, 2024). Samples spanned 15 species across three taxonomic families: Anatidae (ducks), Alcidae (auks) and Laridae (gulls) (Fig. 1a). Venipuncture was performed via the jugular, brachial or medial metatarsal vein by trained personnel (Fig. 1b). Approximately 2 to 3 ml of blood was collected from each bird. Nobuto blood filter strips (Advantec MFS, Inc., USA, Product # 49010010) were saturated with whole blood (approximately 0.1 ml) (Fig. 1b), and the remaining blood was transferred to a centrifuge tube. Samples were kept cool during field activities. At the end of each day, blood samples were centrifuged at 3000 × *g* for 10 minutes to separate serum. The samples were then removed and stored at −20°C until analysis. Filter strips were air-dried in paper coin envelopes (Fig. 1b) and stored at −20°C. For a subset of birds, a second filter strip was collected, dried similarly and stored at room temperature. This work was carried out under the guidelines specified by the Canadian Council on Animal Care with approved protocol 20-05-AL from Memorial University’s Institutional Animal Care Committee, biosafety permit S-103 from Memorial University’s Institutional Biosafety Committee and banding and sampling operations under Federal Bird Banding and Scientific Research Permits (10559, 10851, 10847) as well as ECCC Animal Care Committee approvals (23RR01, 24RR01, 23CS03, 23GR01, 24GR01).

### Sample preparation and laboratory testing

#### Serum

The detection of antibodies against IAV NP was performed using the IDEXX AI MultiS Screen Ab test (IDEXX Canada, Product # 99-12119) at the Memorial University of Newfoundland (MUN) and at the National Wildlife Research Center (NWRC), as per manufacturer’s instructions (Brown *et al.,* 2009). The IDEXX assay is designed to measure the relative level of antibodies against NP for multiple species in avian serum and blood. The assay is performed in 96-well plates that have been coated with IAV NP antigen. Each well requires 100 μl of diluted sample (diluted 1:10 with sample diluent); 10 μl of serum was diluted in 90 μl of sample diluent for the assay. A sample-to-negative (S/N) value of <0.5 was considered positive.

For comparison purposes, a subset of serum samples was also sent to the NCFAD, Canada’s reference laboratory for foreign animal diseases, for the detection of anti-NP antibodies. Detection of anti-NP antibodies was performed following a previously published competitive ELISA (cELISA) (Yang *et al.,* 2008) with minor modifications. Briefly, the optimum working concentrations of the recombinant NP proteins, NP mAb, and horseradish peroxidase (HRP)-labelled goat anti-mouse IgG conjugate, and test serum dilution were predetermined by checkerboard style titrations. The non-treated microtiter plates (Thermo Scientific™, Rochester, NY, USA; Catalog#269620) were coated with 100 μl per well of a predetermined concentration of recombinant NP protein in carbonate buffer (pH 9.6) at 4°C overnight. After washing with phosphate-buffered saline (PBS) plus 0.05% Tween 20 (PBS-T), each well was blocked with 100 μl of 3% heat-inactivated fetal bovine serum (FBS) in PBS plus 0.05% Tween 20 (PBS-T) and incubated for 1 h at 37°C. Then equal volumes (50 μl) of diluted test sera (1:10) and predetermined concentration of NP mAB F28-73 were added to the plates followed by incubation at 37°C for 1 h with agitation; each well requires 5 μl of non-diluted serum to conduct the test (10 μl per sample for duplicate wells). Wells containing competitive mAb mixed with standard IAV-positive sera, negative sera or no serum were utilized as a positive control, negative control or dilution control (DC), respectively. Then HRP-conjugated goat anti-mouse IgG (Jackson Immunoresearch Laboratories, West Grove, PA, USA) was added followed by incubation for 1 h at 37°C with subsequent washing. 3,3,5,5-Tetramethylbenzidine as a peroxidase substrate (TMB) (Sigma-Aldrich, St. Louis, MO, USA) was added and colour development was stopped after 15 min with 50 μl per well of 2.0 M H_2_SO_4_. Colorimetric development was quantified spectrophotometrically at 450 nm with a Molecular Devices Emax precision microplate reader (Molecular Devices, LLC, San Jose, CA, USA). Results were expressed as a percentage of inhibition (PI) and derived by the following formula: PI = (1 – [OD450 of serum sample/OD450 of DC]) × 100. A PI value ≥30% was considered positive.

All samples that were positive for anti-NP antibodies at MUN, NWRC or NCFAD were tested at NCFAD for antibodies against the H5 subtype according to previously described methods (Hochman *et al.,* 2023). Similarly, as described for NP cELISA, the optimum working concentrations of the recombinant H5 proteins, H5 mAb and HRP-labelled anti-mouse conjugate and test serum dilution were predetermined by checkerboard style titrations. The recombinant H5 protein was diluted in a 0.06 M carbonate buffer (pH 9.6), and 100 μl was used per well to coat non-treated 96-well microtiter plates overnight at 4°C. The plates were then washed five times with PBS-T. Each well was blocked with 100 μl of 3% heat-inactivated FBS in PBS-T and incubated for 1 h at 37°C. After washing five times with PBS-T, equal volumes (50 μl) of test serum samples and competitive H5 mAb were mixed and incubated simultaneously at 37°C for 1 h; each well requires 5 μl of non-diluted serum to conduct the test (10 μl per sample for duplicate wells). Wells containing competitive mAb mixed with standard anti-H5 sera, negative sera or no serum were utilized as a positive control, negative control or DC, respectively. Each serum sample was tested in duplicate. After five washes, 100 μl of HRP-labelled goat anti-mouse IgG conjugate was added followed by incubation for 1 h at 37°C. The wells were then washed five times and TMB added as a peroxidase substrate. The reaction was carried out for 15 min at room temperature, followed by addition of 50 μl of 2 M H_2_SO_4_ to each well to stop the reaction. The OD was determined at 450 nm on an automated plate reader as described above for the NP cELISA. Results were interpreted as the PI as described above for the NP cELISA formula. A PI value ≥35% was considered positive.

Validation of H7 cELISA (unpublished data) followed the same principle used for that of H5 cELISA as mentioned above. The procedure for H7 cELISA was the same as for H5 cELISA except that the mAb was H7 mAb (F39AIH7N1-16, Yang *et al.,* 2010), and the coating antigen was recombinant North American H7 HA protein. Their optimal concentrations for H7 cELISA were determined by a checkerboard-style titration. Results were interpreted as the PI calculated according to the following formula: % inhibition = (1 – [OD450 of serum sample/OD450 of DC]) × 100. A PI value ≥35% was considered positive.

#### Filter strips

Filter strips saturated with whole blood were cut and submerged into 400 μl of PBS (pH 7.2) (Gibco™, USA, Product # 20012027) at 4°C for 24 hours. The eluate was then transferred to new tubes and used for detection of anti-NP antibodies with the IDEXX AI MultiS Screen Ab test, as described for serum, without initial dilution.

As above, a subset of eluate samples was also sent to NCFAD for anti-NP antibody detection. All anti-NP antibody positive eluates were tested for anti-H5 antibodies at NCFAD.

#### Statistical analysis

All analyses were conducted in R version 4.2.2 (‘Innocent and Trusting’) (R Core Team, 2024) using RStudio ‘Mountain Hydrangea’ Release (547dcf86, 2023-07-07) (Posit team, 2024). All analyses were conducted on the subset of samples that had paired results across laboratories and species.

To evaluate quantitative data, Bland–Altman plots (Bland and Altman, 1986) were generated to visualize agreement between S/N control ratios or PI values from serum and filter strip eluate samples. Spearman rank correlation (Spearman, 1904; Caruso and Cliff, 1997) was calculated using the base R *cor.test*() function to assess the strength and direction of the monotonic relationship between S/N or PI values across sample types. Exact McNemar’s test (McNemar, 1947), using the *exact2x2*() function from the exact 2 × 2 package in R (Fay *et al.,* 2009), was applied to compare discordant pairs between filter strips stored at room temperature and frozen, specifically assessing the impact of storage conditions on performance.

For binary outcomes, manufacturer/reference laboratory thresholds were used to classify serum samples as positive or negative for both anti-NP, anti-H5 tests and anti-H7 tests. Sensitivity, specificity and percentage agreement were calculated for filter strip eluate samples using these thresholds, with serum results serving as the gold standard. Receiver operating characteristic (ROC) curves (Hanley and McNeil, 1982) were generated using the *roc*() function from the package pROC (Robin *et al.,* 2011), to plot sensitivity against 1 − specificity across different thresholds. The pROC package was also used for the Youden index (Youden, 1950), employed to determine the optimal threshold that maximizes the sum of sensitivity and specificity, balancing diagnostic accuracy.

## Results

### Performance of filter strips saturated with whole blood stored frozen versus serum for the detection of anti-NP and anti-H5 antibodies

#### NP—IDEXX (frozen filter strips vs. serum)

**Figure 2:**
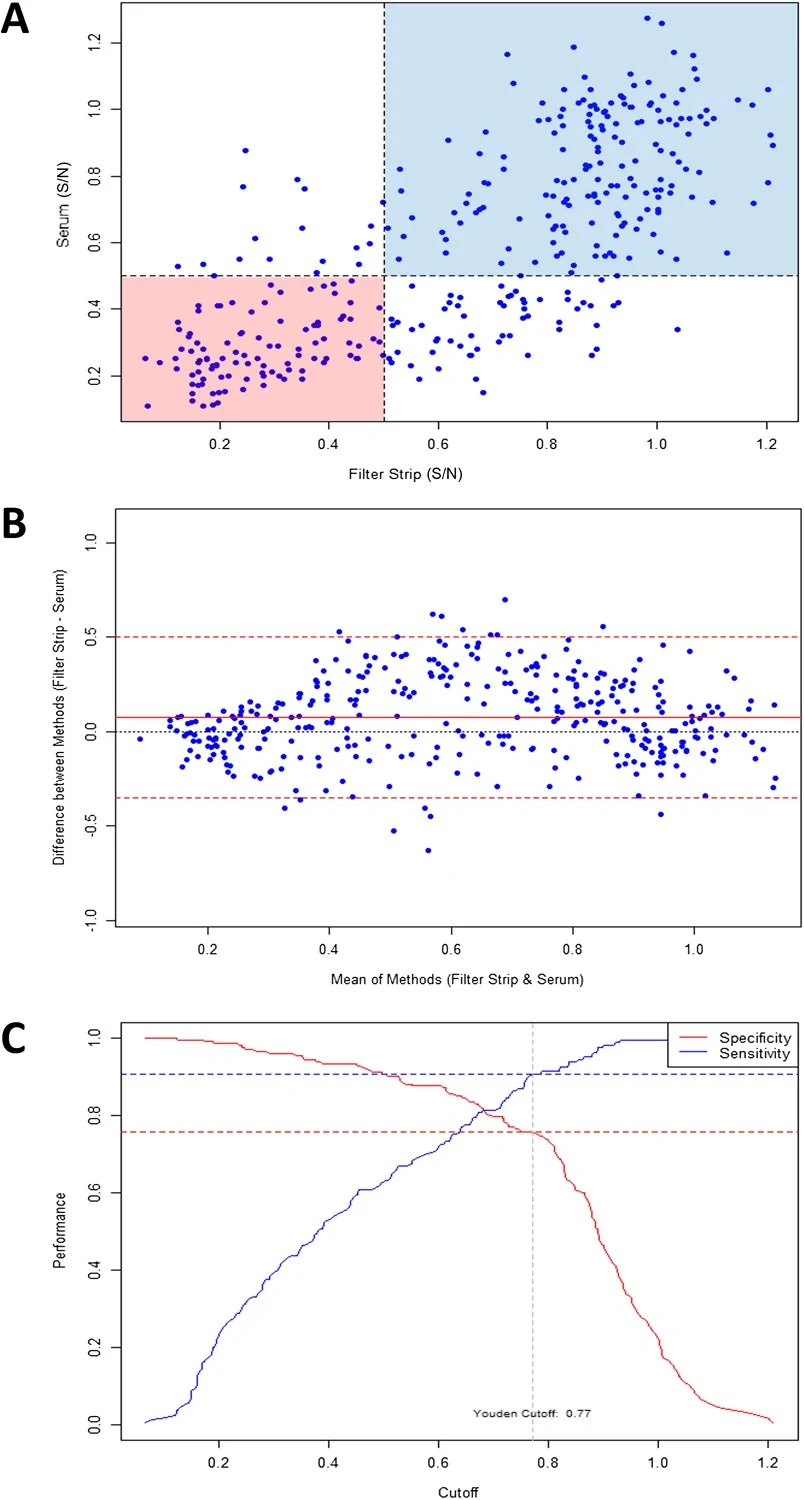
**Analysis of S/N values for NP antibody detection using IDEXX AI MultiS Screen Ab ELISA in serum and filter strips stored frozen.** (**a**) Scatter plot showing the correlation between S/N values, with dashed black lines representing the original threshold value. The quadrant where both tests are positive is shaded in red (bottom left), while the quadrant where both are negative is shaded in blue (top right). (**b**) Bland–Altman plot comparing S/N values, with the solid red line representing the mean difference (bias) and dashed red lines indicating the limits of agreement (mean difference ± 1.96 SD), while the black dashed line at *y* = 0 serves as a reference. (**c**) ROC curve illustrating the diagnostic performance of filter strips relative to serum, with sensitivity (blue line) and specificity (red line) plotted against different cutoff thresholds. The grey dashed vertical line marks the optimal cutoff identified by the Youden index, with horizontal dashed lines indicating the corresponding sensitivity (blue) and specificity (red) at this cutoff.

A total of 352 paired serum and whole blood filter strip samples stored frozen were analysed to assess agreement for anti-NP antibody detection using the IDEXX ELISA (Table 1). Testing was conducted using the same protocol at both NWRC (*n* = 168) and MUN (*n* = 184). Spearman’s rank correlation demonstrated a positive relationship between serum and whole blood filter strip values (*ρ* = 0.76) (Table 1 and Fig. 2a). The Bland–Altman plot shows a positive mean difference, indicating that filter strips yielded higher S/N ratios than serum, resulting in fewer positive detections when using the original threshold (S/N < 0.5) (Table 1 and Fig. 2b). The performance of whole blood on filter strips was evaluated using both the original manufacturer threshold and the Youden index-optimized threshold (Fig. 2c). The optimized threshold of <0.7708 resulted in overall improved sensitivity compared to the original threshold, with a corresponding decrease in specificity, leading to better overall agreement with serum (Table 1 and Fig. 2c).

**Table 1:** Comparison of anti-NP, anti-H5, and anti-H7 avian influenza virus antibody detection in serum stored frozen and filter strips stored at room temperature and frozen using different ELISA methodologies

		Gold standard			Method compared				Number of samples
Target	Test	Sample	Storage	Cutoff	Sample	Storage	Original cutoff	Youden cutoff	Total (ECCC/MUN)
NP	IDEXX	Serum	Frozen	< 0.50	Filter Strip	Frozen	< 0.50	< 0.77	352 (168/184)
NP	IDEXX	Serum	Frozen	< 0.50	Filter Strip	Room Temperature	< 0.50	< 0.83	66 (66/0)
NP	NCFAD	Serum	Frozen	≥ 30.00	Filter Strip	Frozen	≥ 30.00	> 39.56	127 (124/3))
H5	NCFAD	Serum	Frozen	≥ 35.00	Filter Strip	Frozen	≥ 35.00	> 20.37	194 (157/37)
H5	NCFAD	Serum	Frozen	≥ 35.00	Filter Strip	Room Temperature	≥ 35.00	> 22.09	37 (37/0)
H7	NCFAD	Serum	Frozen	≥ 35.00	Filter Strip	Frozen	≥ 35.00	> −3.73	120 (120/0)

**Table 1:** Continued.

Spearman’s rank correlation		Bland–Altman		Original cutoff			Youden cutoff		
rho	p-value	Mean Difference	Limits of Agreement	% Agreement	Sensitivity	Specificity	% Agreement	Sensitivity	Specificity
0.76	< 0.001	0.07	−0.35, 0.50	78.13	63.13	90.63	82.39	90.63	75.52
0.71	< 0.001	0.28	−0.13, 0.69	59.09	20.59	100.00	84.84	73.53	96.88
0.58	< 0.001	−11.43	−60.26, 37.39	82.68	86.84	46.15	74.02	72.81	84.62
0.65	< 0.001	−20.69	−60.23, 18.85	56.70	29.31	97.44	80.41	75.00	88.46
0.44	0.007	−14.81	−62.21, 32.59	51.35	10.00	100.00	78.38	90.00	64.71
0.14	0.120	−8.31	−51.46, 34.84	88.33	0.00	100.00	29.17	21.50	92.31

#### NP—NCFAD (frozen filter strips vs. serum)

**Figure 3:**
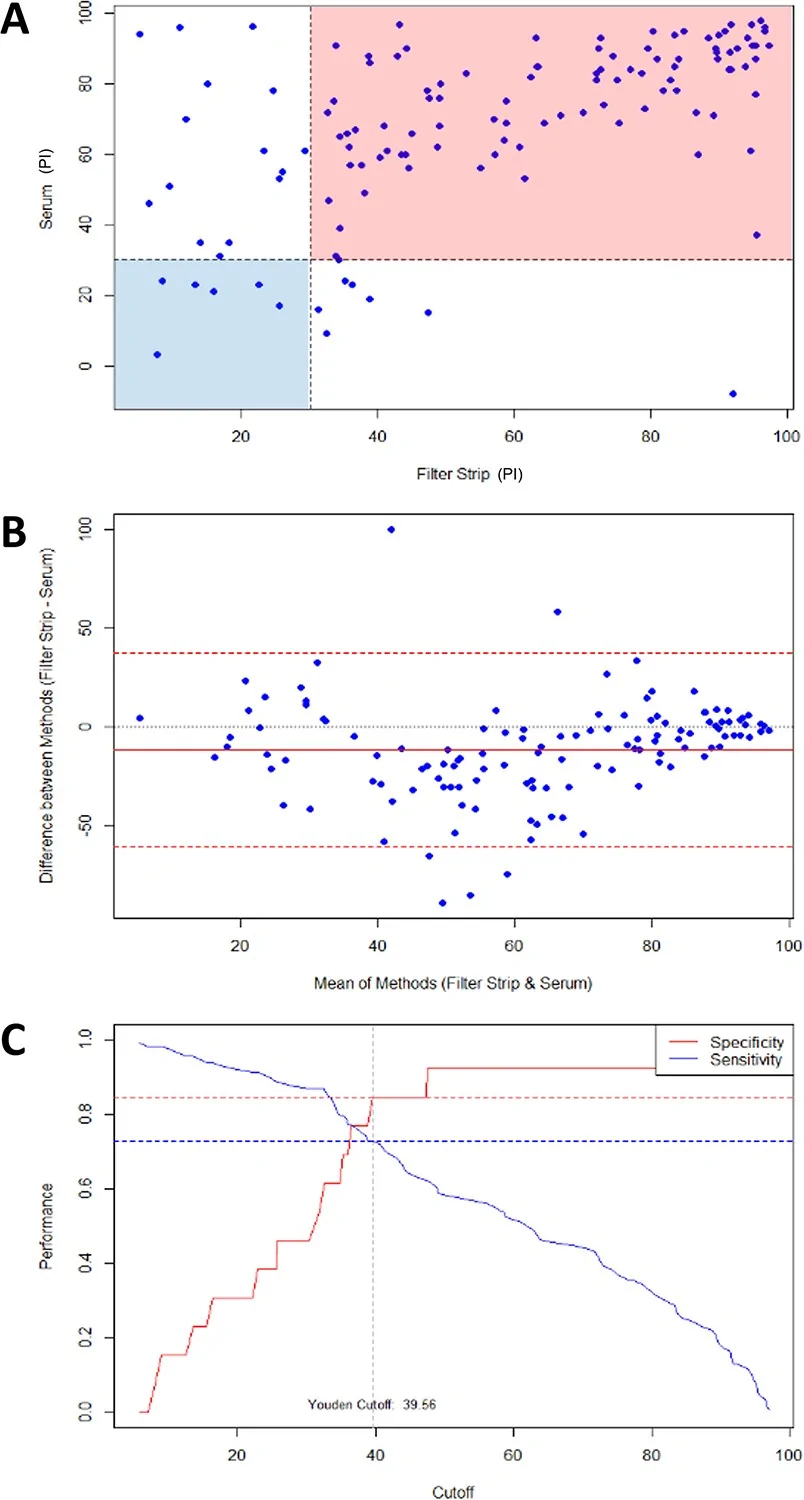
**Analysis of PI values for NP antibody detection using NCFAD in-house ELISA in serum and filter strips stored frozen**. (**a**) Scatter plot showing the correlation between PI values, with dashed black lines representing the original threshold value. The quadrant where both tests are positive is shaded in red (top right), while the quadrant where both are negative is shaded in blue (bottom left). (**b**) Bland–Altman plot comparing PI values, with the solid red line representing the mean difference (bias) and dashed red lines indicating the limits of agreement (mean difference ± 1.96 SD), while the black dashed line at *y* = 0 serves as a reference. (**c**) ROC curve illustrating the diagnostic performance of filter strips relative to serum, with sensitivity (blue line) and specificity (red line) plotted against different cutoff thresholds. The grey dashed vertical line marks the optimal cutoff identified by the Youden index, with horizontal dashed lines indicating the corresponding sensitivity (blue) and specificity (red) at this cutoff.

For anti-NP antibody detection using the NCFAD ELISA, 127 paired serum and whole blood filter strip samples stored frozen were analysed (Table 1). The samples originated from both NWRC (*n* = 124) and MUN (*n* = 3) laboratories, but all samples were tested at NCFAD using the same protocol. For the subset sent from NWRC, this included all samples collected, not just those that tested positive at NWRC. Spearman’s rank correlation showed a positive relationship, albeit weaker correlation compared to IDEXX, between serum and whole blood on filter strip values (*ρ* = 0.58) (Table 1 and Fig. 3a). The Bland–Altman plot revealed a negative mean difference, indicating that in general, whole blood on filter strips produced lower PI values compared to serum, leading to fewer positive detections when using the original threshold (PI > 30) (Table 1 and Fig. 3b). However, the limits of agreement were wide, reflecting substantial variability when measuring PI values between sample types (Table 1 and Fig. 3b). The original threshold resulted in poor specificity, with values falling below 50% (Table 1). The Youden index-optimized threshold of >39.56 improved specificity, with a reduction in sensitivity and percentage agreement (Table 1 and Fig. 3c).

#### H5—NCFAD (frozen filter strips vs. serum)

**Figure 4:**
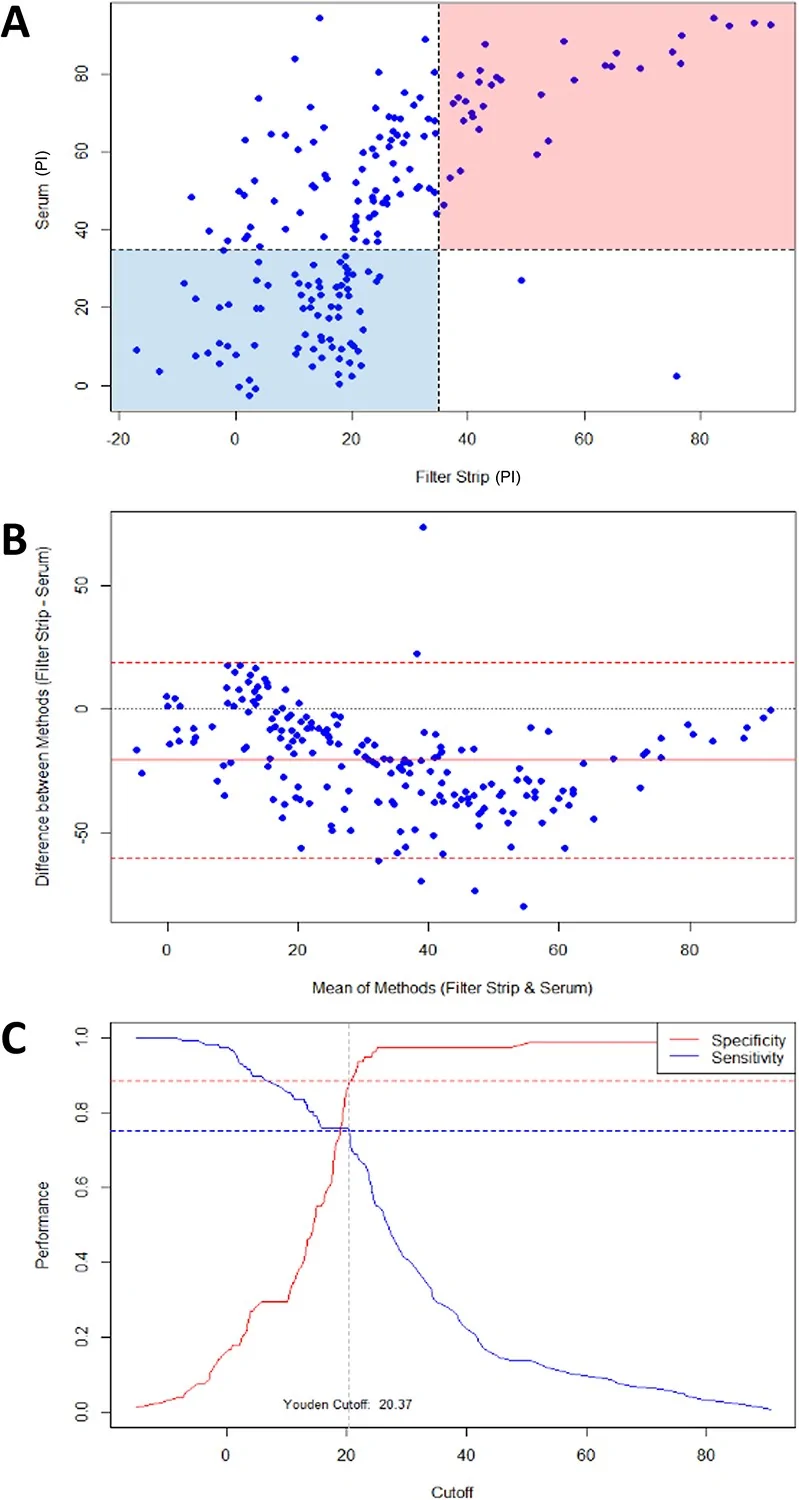
**Analysis of PI values for H5 antibody detection using NCFAD in-house ELISA in serum and filter strips stored frozen.** (**a**) Scatter plot showing the correlation between PI values, with dashed black lines representing the original threshold value. The quadrant where both tests are positive is shaded in red (top right), while the quadrant where both are negative is shaded in blue (bottom left). (**b**) Bland–Altman plot comparing PI values, with the solid red line representing the mean difference (bias) and dashed red lines indicating the limits of agreement (mean difference ± 1.96 SD), while the black dashed line at *y* = 0 serves as a reference. (**c**) ROC curve illustrating the diagnostic performance of filter strips relative to serum, with sensitivity (blue line) and specificity (red line) plotted against different cutoff thresholds. The grey dashed vertical line marks the optimal cutoff identified by the Youden index, with horizontal dashed lines indicating the corresponding sensitivity (blue) and specificity (red) at this cutoff.

A total of 194 paired serum and whole blood filter strip samples stored frozen were analysed to assess agreement for H5 antibody detection using the NCFAD ELISA (Table 1). The samples originated from NWRC (*n* = 157) and MUN (*n* = 37), and all were tested using the same protocol at NCFAD. Spearman’s rank correlation demonstrated a positive relationship between serum and filter strip values (*ρ* = 0.65) (Table 1 and Fig. 4a). The Bland–Altman plot showed a negative mean difference, indicating that whole blood on filter strips yielded lower PI values than serum, leading to fewer positive detections when using the original threshold (PI > 35) (Table 1 and Fig. 4b). However, the limits of agreement were wide, reflecting substantial variability when measuring PI values between sample types (Table 1 and Fig. 4b). The original threshold resulted in high specificity but very poor sensitivity (Table 1). The Youden index-optimized threshold of >20.37 increased sensitivity while decreasing specificity, leading to a more balanced diagnostic performance (Table 1 and Fig. 4c).

#### H7—NCFAD (frozen filter strips vs. serum)

A total of 120 paired serum and filter strip samples stored frozen were analysed for H7 antibody detection using the NCFAD ELISA (Table 1). The samples originated from NWRC, and all were tested following the same protocol at NCFAD. Spearman’s rank correlation showed a weak positive relationship between serum and filter strip values (*ρ* = 0.14) (Table 1 and Supplemental Fig. S3a). The Bland–Altman plot revealed a negative mean difference, with wide limits of agreement, reflecting significant variability and indicating that filter strips tended to yield lower PI values than serum (Table 1 and Supplemental Fig. S3b). The original threshold resulted in no sensitivity and poor overall performance (Table 1 and Supplemental Fig. S3c). The Youden index-optimized threshold provided some improvement in sensitivity, but it remained low (Table 1 and Supplemental Fig. S3c).

### Impact of storage condition (room temperature versus frozen) on the performance of filter strip samples in detecting anti-NP and anti-H5 IAV antibodies

#### NP—IDEXX (room temperature filter strips vs. serum)

The same analyses were conducted for a subset of samples stored at room temperature (*n* = 66), all tested at NWRC (Table 2). The agreement between serum and whole blood on filter strips was lower (*ρ* = 0.71), and Bland–Altman analysis revealed a greater mean difference, indicating that room temperature storage further reduced the detection sensitivity of whole blood on filter strips (Table 1 and Supplemental Fig. S1a and b). The Youden index-optimized threshold for room temperature filter strips was higher than that for frozen filter strips at <0.8274 but similarly improved sensitivity, with a reduction in specificity, for better overall agreement with serum (Table 1 and Supplemental Fig. S1c).

#### H5—NCFAD (room temperature filter strips vs. serum)

The same analyses were conducted for a subset of samples stored at room temperature (*n* = 37), originating from NWRC and tested at NCFAD. The agreement between serum and whole blood on filter strips was lower (*ρ* = 0.44) than whole blood on filter strips stored frozen (Table 1 and Supplemental Fig. S2a), with Bland–Altman analysis revealing a smaller mean difference but broader limits of agreement compared to the frozen samples (Table 1 and Supplemental Fig. S2b). The Youden index-optimized threshold for room temperature samples, set at >22.09, improved sensitivity while reducing specificity, leading to better overall agreement with serum (Table 1 and Supplemental Fig. S2c).

### Comparison of frozen versus room temperature storage of filter strips for anti-NP and anti-H5 antibody detection

**Figure 5:**
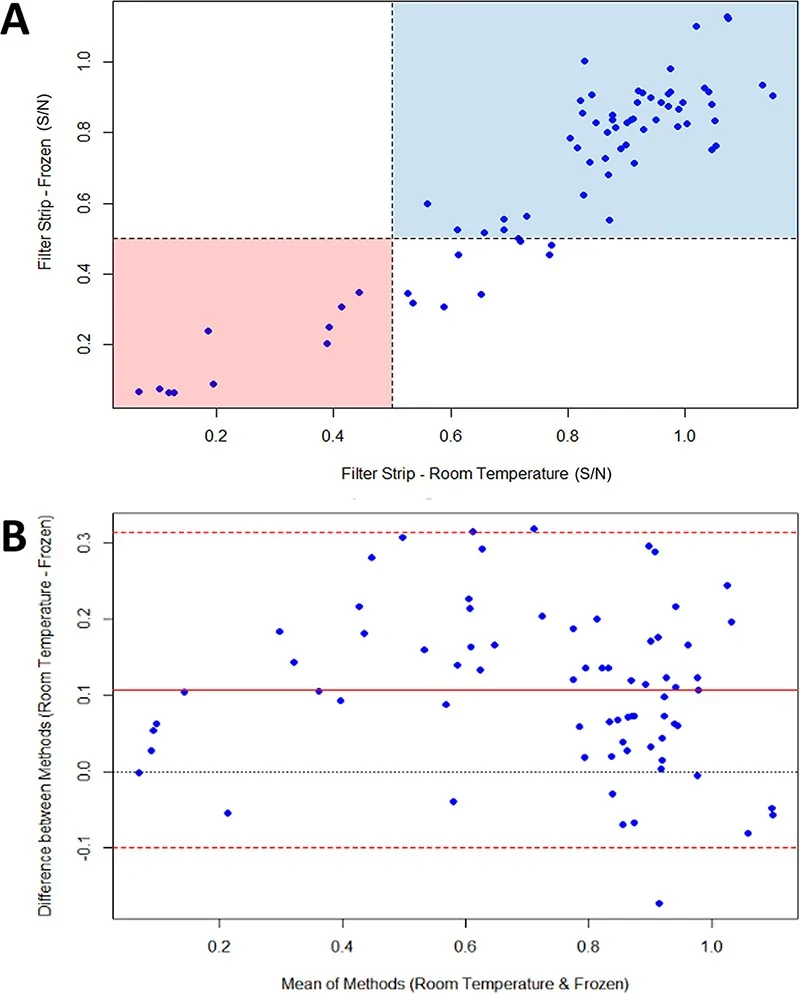
**Analysis of S/N values for NP antibody detection using IDEXX AI MultiS Screen Ab ELISA in filter strips stored frozen and at room temperature**. (**a**) Scatter plot showing the correlation between S/N values, with dashed black lines representing the original threshold value. The quadrant where both tests are positive is shaded in red (bottom left), while the quadrant where both are negative is shaded in blue (top right). (**b**) Bland–Altman plot comparing S/N values, with the solid red line representing the mean difference (bias) and dashed red lines indicating the limits of agreement (mean difference ± 1.96 SD), while the black dashed line at *y* = 0 serves as a reference.

**Figure 6:**
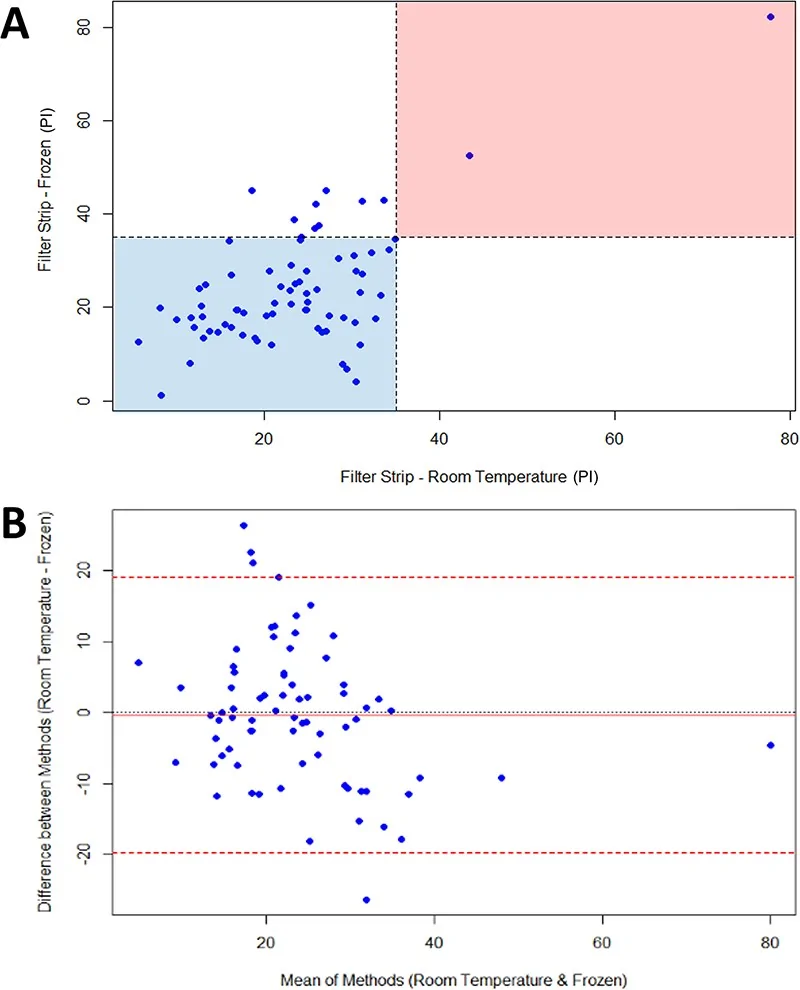
**Analysis of PI values for H5 antibody detection using NCFAD in-house ELISA in filter strips stored frozen and at room temperature.** (**a**) Scatter plot showing the correlation between PI values, with dashed black lines representing the original threshold value. The quadrant where both tests are positive is shaded in red (top right), while the quadrant where both are negative is shaded in blue (bottom left). (**b**) Bland–Altman plot comparing PI values, with the solid red line representing the mean difference (bias) and dashed red lines indicating the limits of agreement (mean difference ± 1.96 SD), while the black dashed line at *y* = 0 serves as a reference.

A total of 73 paired filter strip samples (one stored frozen and one stored at room temperature) were analysed to assess the effect of storage conditions on anti-NP and anti-H5 antibody detection, all tested at NWRC (Table 2). High percentage agreement was observed between room temperature and frozen storage, with Spearman’s rank correlation indicating a stronger positive correlation for NP detection (*ρ* = 0.84) than for H5 detection (*ρ* = 0.38) (Table 2 and Figs 5a and 6a).

The Bland–Altman plot for anti-NP antibody detection using the IDEXX ELISA showed a positive mean difference, indicating that filter strips stored at room temperature yielded higher S/N ratios than those stored frozen, resulting in fewer positive detections (Table 2 and Fig. 5b). For H5 detection using the NCFAD ELISA, the Bland–Altman plot showed a negative mean difference, with room temperature storage producing lower PI values, resulting in fewer positive detections (Table 2 and Fig. 6b). The wide limits of agreement for H5 detection suggest variability in detection performance across different storage conditions (Table 2 and Fig. 6b). Exact McNemar’s test confirmed statistically significant differences in performance between storage conditions, with an odds ratio of infinity, indicating that frozen samples resulted in more positive detections, with no discordant pairs favouring room temperature (Table 2).

**Table 2:** Comparison of anti-NP and anti-H5 avian influenza virus antibody detection using filter strips stored at room temperature versus frozen

		Method 1			Method 2			Number of samples	
Target	Test	Sample	Cutoff	Storage	Sample	Cutoff	Storage	Total (ECCC/MUN)	% Agreement
NP	IDEXX	Filter Strip	< 0.50	Frozen	Filter Strip	< 0.5	Room Temperature	73 (73/0)	87.67
HS	NCFAD	Filter Strip	> 35.00	Frozen	Filter Strip	> 35.00	Room Temperature	73 (73/0)	89.04

**Table 2:** Continued.

Spearman’s Rank Correlation		Bland–Altman		Exact McNemar				
rho	p-value	Mean Difference	Limits of Agreement	# discordant pairs (b1)	# discordant pairs (c2)	p-value	Odds ratio	95% Confidence interval
0.84	< 0.001	0.11	−0.01, 0.31	9	0	0.004	Inf	1.97, Inf
0.38	< 0.001	−0.38	−19.83, 19.07	8	0	0.008	Inf	1.71, Inf

### Taxonomic order, family and species variability in filter strip performance

#### NP—NCFAD (frozen filter strips vs. serum)

Comparison of PI values for anti-NP antibodies detected using the NCFAD in-house ELISA (Table 3) shows that serum consistently produced higher PI values than filter strips for positive samples, with variability observed across species. In positive samples from Anseriformes (*n* = 111), the overall mean PI for serum (74.19%) was higher than that of frozen whole blood on filter strips (61.57%), with filter strips exhibiting a wider range (6.5–97.17%) compared to serum (30–98%). Mallards (*n* = 94), the largest sample size in this group, showed the closest alignment between mean PI values for serum (74.77%) and frozen whole blood on filter strips (62.96%), while American Black Ducks (*n* = 15) followed a similar trend with lower mean PI values for filter strips (53.81%) compared to serum (71.40%). For negative Anseriformes samples (*n* = 13), filter strips had a higher overall mean PI (31.28%), exceeding the 30% threshold, and exhibited a wider range (7.63–92.04%), suggesting potential misclassification of negatives as positives.

**Table 3:** Comparison of PI from paired serum (gold standard) and filter strips stored frozen, tested with NCFAD in-house ELISA for anti-NP avian influenza antibodies, summarized by taxonomic order, family, and species

				Gold standard (serum)			Method compare (filter strip)
Order	Family	Species	Sample size	Result	No.	Mean PI% (min, max)	Mean PI% (min, max)
Anseriformes			124	Positive	111	74.19 (30.00, 98.00)	61.57 (6.50, 97.17)
				Negative	13	16.08 (−8.00, 24.00)	31.28 (7.63, 92.04)
	Anatidae		124	Positive	111	74.19 (30.00, 98.00)	61.57 (6.50, 97.17)
				Negative	13	16.08 (−8.00, 24.00)	31.28 (7.63, 92.04)
		American Black Duck	17	Positive	15	71.40 (39.00, 88.00)	53.81 (14.96, 95.20)
				Negative	2	15.50 (15.00, 16.00)	39.34 (31.29, 47.39)
		Mallard	104	Positive	94	74.77 (30.00, 98.00)	62.96 (6.50, 97.17)
				Negative	10	16.90 (−8.00, 24.00)	29.55 (7.63, 92.04)
		Northern Pintail	1	Positive	1	74.00 (74.00, 74.00)	73.09 (73.09, 73.09)
				Negative	0	–	–
		Wood Duck	2	Positive	1	62.00 (62.00, 62.00)	35.81 (35.81, 35.81)
				Negative	1	9.00 (9.00, 9.00)	32.46 (32.46, 32.46)
Charadriiformes			3	Positive	3	95.41 (94.05, 96.22)	12.56 (5.06, 21.63)
				Negative	0	–	–
	Alcidae		3	Positive	3	95.41 (94.05, 96.22)	12.56 (5.06, 21.63)
				Negative	0	–	–
		Atlantic Puffin	3	Positive	3	95.41 (94.05, 96.22)	12.56 (5.06, 21.63)
				Negative	0	–	–
Total			127	Positive	114	74.75 (30.00, 98.00)	60.28 (5.06, 97.17)
				Negative	13	16.08 (−8.00, 24.00)	31.28 (7.63, 92.04)

In Charadriiformes, represented solely by Atlantic Puffins (*n* = 3), mean PI values were higher for serum (95.41%) compared to filter strips (12.56%), though the small sample size limits conclusions about performance in this group.

#### NP—IDEXX (frozen filter strips vs. serum)

Table 4 presents S/N for anti-NP antibodies tested with the IDEXX Multi-S ELISA. For Anseriformes positive samples (*n* = 103), serum had a mean S/N value of 0.32, while filter strips had a mean S/N of 0.5, which is the same as the original threshold. Frozen filter strips with whole blood exhibited a broader range of S/N values (0.06–1.04) compared to serum (0.11–0.47). Among Anseriformes, American Black Ducks, Mallards and Wood Ducks demonstrated greater differences between mean S/N values for serum and filter strips, with filter strip means nearing or exceeding the threshold for certain individuals. For negative Anseriformes samples (*n* = 65), all species had mean S/N values above the 0.5 threshold for both serum and filter strips, though filter strips showed a higher overall mean (0.83 vs. 0.71).

**Table 4:** Comparison of S/N Ratios from paired serum (gold standard) and filter strip samples stored frozen, tested with IDEXX multi-S ELISA for anti-NP avian influenza antibodies, summarized by taxonomic order, family, and species

				Gold standard (serum)			Method compare (filter strip)
Order	Family	Species	Sample size	Result	No.	Mean S/N (min, max)	Mean S/N (min, max)
Anseriformes			168	Positive	103	0.32 (0.11, 0.47)	0.50 (0.06, 1.04)
				Negative	65	0.71 (0.50, 1.06)	0.83 (0.19, 1.13)
	Anatidae		168	Positive	103	0.32 (0.11, 0.47)	0.50 (0.06, 1.04)
				Negative	65	0.71 (0.50, 1.06)	0.83 (0.19, 1.13)
		American Black Duck	14	Positive	8	0.32 (0.21, 0.41)	0.57 (0.16, 0.89)
				Negative	6	0.66 (0.56, 0.82)	0.76 (0.50, 0.94)
		Green-Winged Teal	8	Positive	3	0.25 (0.19, 0.31)	0.30 (0.09, 0.48)
				Negative	5	0.73 (0.57, 1.06)	0.86 (0.73, 1.10)
		Mallard	130	Positive	85	0.32 (0.17, 0.47)	0.50 (0.06, 1.04)
				Negative	45	0.68 (0.50, 1.06)	0.81 (0.19, 1.03)
		Northern Pintail	4	Positive	2	0.32 (0.28, 0.35)	0.36 (0.34, 0.37)
				Negative	2	0.57 (0.56, 0.57)	1.02 (0.90, 1.13)
		Wood Duck	12	Positive	5	0.21 (0.11,0.34)	0.58 (0.07, 0.82)
				Negative	7	0.95 (0.77, 1.03)	0.90 (0.87, 0.92)
Charadriiformes			184	Positive	57	0.28 (0.11, 0.49)	0.31 (0.12, 0.90)
				Negative	127	0.86 (0.51, 1.28)	0.85 (0.12, 1.21)
	Alcidae		34	Positive	18	0.22 (0.11, 0.41)	0.24 (0.15, 0.44)
				Negative	16	0.80 (0.51, 1.17)	0.88 (0.38, 1.21)
		Atlantic Puffin	17	Positive	3	0.22 (0.16, 0.30)	0.34 (0.24, 0.44)
				Negative	14	0.83 (0.54, 1.17)	0.94 (0.55, 1.21)
		Common Murre	17	Positive	15	0.22 (0.11, 0.41)	0.23 (0.15, 0.36)
				Negative	2	0.58 (0.51, 0.65)	0.43 (0.38, 0.48)
	Laridae		150	Positive	39	0.31 (0.12, 0.49)	0.33 (0.12, 0.90)
				Negative	111	0.87 (0.53, 1.28)	0.84 (0.12, 1.21)
		Black-Legged Kittiwake	9	Positive	5	0.38 (0.30, 0.44)	0.56 (0.38, 0.73)
				Negative	4	0.77 (0.74, 0.83)	0.99 (0.94, 1.02)
		Glaucous Gull	2	Positive	1	0.23 (0.23, 0.23)	0.19 (0.19, 0.19)
				Negative	1	0.64 (0.64, 0.64)	0.51 (0.51, 0.51)
		Great Black-Backed Gull	17	Positive	9	0.23 (0.17, 0.30)	0.17 (0.15, 0.25)
				Negative	8	0.79 (0.53, 0.96)	0.54 (0.12, 0.93)
		Herring Gull	122	Positive	24	0.33 (0.12, 0.49)	0.35 (0.12, 0.90)
				Negative	98	0.89 (0.54, 1.28)	0.87 (0.24, 1.21)
Total			352	Positive	160	0.30 (0.11, 0.49)	0.43 (0.06, 1.04)
				Negative	192	0.81 (0.50, 1.28)	0.84 (0.12, 1.21)

For positive samples from Charadriiformes (*n* = 57), serum and whole blood on filter strips had similar overall mean S/N values (0.28 and 0.31, respectively). Filter strips showed a wider range (0.12–0.90) compared to serum (0.11–0.49). Within the Alcidae, positive samples for Atlantic Puffins and Common Murres demonstrated good alignment between mean S/N values for serum and filter strip samples, with both the mean and range remaining below the threshold of 0.5. For the Alcidae family, negative samples showed good alignment between mean S/N values for serum and filter strip samples, except for Common Murres, although the sample size was only two. Among positive samples from the Laridae, mean S/N values for filter strips and serum were similar across species, except for Black-Legged Kittiwakes, where the filter strip mean (0.56) exceeded the threshold of 0.5, resulting in false negatives. For Herring Gulls, while the mean S/N for filter strips (0.35) was below the threshold, the broader range (0.12 to 0.90) indicated additional false negatives. For negative samples, mean S/N values for serum and filter strips were closely aligned, with false positives observed only in Great Black-Backed Gulls and Herring Gulls.

#### H5—NCFAD (frozen filter strips vs. serum)

PI values for anti-H5 antibodies detected using the NCFAD in-house ELISA are summarized in Table 5. Among Anseriformes positive samples (*n* = 97), serum had a mean PI of 61.09%, whereas filter strips showed a lower mean of 28.61%, below the original threshold cutoff of 35%, with a broader range (−7.63% to 92.08%) compared to serum (35.75% to 94.48%). For all Anseriformes species, the mean PI for filter strips was below the threshold, except for Green-Winged Teal (*n* = 5), which showed a mean PI of 41.83%. Negative Anseriformes samples (*n* = 60) showed better consistency between sample types, with mean PI values of 17.64% for serum and 15.56% for filter strips*.*

**Table 5:** Comparison of PI from paired serum (gold standard) and filter strip samples stored frozen, tested with NCFAD in-house ELISA for anti-H5 avian influenza antibodies, summarized by taxonomic order, family, and species

				Gold standard (serum)			Method compare (filter strip)
Order	Family	Species	Sample size	Result	No.	Mean PI% (min, max)	Mean PI% (min, max)
Anseriformes			157	Positive	97	61.09 (35.75, 94.48)	28.61 (−7.63, 92.08)
				Negative	60	17.64 (0.38, 34.65)	15.56 (−17.07, 75.96)
	Anatidae		157	Positive	97	61.09 (35.75, 94.48)	28.61 (−7.63, 92.08)
				Negative	60	17.64 (0.38, 34.65)	15.56 (−17.07, 75.96)
		American Black Duck	15	Positive	10	55.08 (37.16, 79.73)	23.61 (−1.33, 40.86)
				Negative	5	17.89 (5.23, 33.35)	16.65 (3.29, 21.70)
		Green-Winged Teal	5	Positive	4	59.93 (36.99, 94.37)	41.83 (22.52, 82.28)
				Negative	1	13.02 (13.02, 13.02)	11.92 (11.92, 11.92)
		Mallard	127	Positive	79	62.05 (35.75, 94.48)	28.81 (−4.50, 92.08)
				Negative	48	18.87 (0.38, 34.65)	15.99 (−13.05, 75.96)
		Northern Pintail	3	Positive	3	58.69 (48.50, 72.47)	22.86 (−7.63, 38.76)
				Negative	0	–	–
		Wood Duck	7	Positive	1	57.00 (57.00, 57.00)	27.20 (27.20, 27.20)
				Negative	6	8.39 (2.91, 12.57)	11.79 (−17.07, 21.00)
Charadriiformes			37	Positive	19	64.91 (37.73, 85.67)	37.48 (1.68, 76.58)
				Negative	18	14.82 (−2.58, 27.07)	2.74 (−8.83, 24.27)
	Alcidae		18	Positive	14	67.82 (38.27, 85.67)	46.76 (6.72, 76.58)
				Negative	4	20.04 (7.56, 26.76)	13.34 (−6.91, 24.27)
		Atlantic Puffin	3	Positive	1	47.48 (47.48, 47.48)	6.72 (6.72, 6.72)
				Negative	2	13.80 (7.56, 20.05)	5.38 (−6.91, 17.66)
		Common Murre	15	Positive	13	69.38 (38.27, 85.67)	49.84 (15.12, 76.58)
				Negative	2	26.28 (25.80, 26.76)	21.30 (18.33, 24.27)
	Laridae		19	Positive	5	56.77 (37.73, 80.59)	11.51 (1.68, 34.34)
				Negative	14	13.32 (−2.58, 27.07)	−0.29 (−8.83, 5.55)
		Black-Legged Kittiwake	2	Positive	0	–	–
				Negative	2	0.29 (−0.93, 1.50)	2.91 (2.44, 3.38)
		Glaucous Gull	1	Positive	1	38.58 (38.58, 38.58)	2.03 (2.03, 2.03)
				Negative	0	–	–
		Great Black-Backed Gull	3	Positive	3	60.26 (37.73, 80.59)	16.49 (1.68, 34.34)
				Negative	0	–	–
		Herring Gull	13	Positive	1	64.50 (64.50, 64.50)	6.05 (6.05, 6.05)
				Negative	12	15.50 (−2.58, 27.07)	−0.83 (−8.83, 5.55)
Total			194	Positive	116	61.71 (35.75, 94.48)	30.07 (−7.63, 92.08)
				Negative	78	16.99 (−2.58, 34.65)	12.60 (−17.07, 75.96)

For the Charadriiformes positive samples, the overall mean PI was greater than the original 35% threshold for both serum (64.91%) and whole blood on filter strips (37.48%). However, the mean PI for filter strips was still quite close to threshold and the range was wider (1.68% to 76.58%) than serum (37.73% to 85.67%). Among the Charadriiformes, there was better alignment between the PI values of the positive samples (*n* = 19) for the Alcidae family (67.82% for serum and 46.76% for filter strips) than the Laridae family (56.77% for serum and 11.51% for filter strips). Among the Alcidae, alignment between the mean PI values for positive samples was reasonable for Common Murres (69.38% for serum and 49.84% for filter strips). Atlantic Puffin and all Laridae sample sizes were low, but alignment in PI values between sample types was poor for both positive and negative samples.

## Discussion

Overall, moderate to strong correlations were observed between the performance of whole blood filter strips and paired serum samples for detecting anti-NP and anti-H5 IAV antibodies across multiple ELISAs (IDEXX and NCFAD). The strength of the correlation varied by the target and ELISA type, with notably poor agreement for anti-H7 antibody detection, which showed weak correlation and low sensitivity.

The original thresholds set by the manufacturers or reference laboratories for both IDEXX and NCFAD ELISAs often resulted in very poor sensitivity when applied to blood collected on filter strips across the taxa tested. This underscores the need for threshold optimization to enhance diagnostic performance of filter strips for whole blood samples collected from wild animals. The goal of threshold optimization is to balance sensitivity and specificity, but this balance depends on the objectives of the surveillance programme.

For the IDEXX ELISA, stronger positive correlations between serum and whole blood on filter strip values were observed compared to NCFAD assay, with smaller mean differences noted in the Bland–Altman plots. Both IDEXX and NCFAD ELISAs target anti-NP antibodies using monoclonal antibodies and have been validated for use in multiple avian species. However, differences observed in this study may reflect logistical factors. Filter strip samples saturated with whole blood were first eluted and tested locally using the IDEXX ELISA, with positive samples subsequently frozen and shipped across the country for confirmatory testing with the NCFAD ELISA. Despite priority overnight shipping, this process inevitably introduced a freeze–thaw cycle and a time delay between sample elution and testing, potentially compromising antibody stability. These logistical factors likely contributed to the lower observed performance of the NCFAD assays for filter strip samples in this study. These findings emphasize the importance of minimizing delays in sample handling and processing after elution, particularly for filter strip samples. Running the full suite of testing in a single laboratory, where feasible, could mitigate these challenges.

Storage conditions notably influenced the performance of filter strips, especially for the H5 ELISA. Freezing or cooling filter strips after blood collection preserves antibody integrity, consistent with findings from studies involving human and animal samples (Mei, 2014; Bevins *et al.,* 2016; Björkesten *et al.,* 2017; Kunkel *et al.,* 2023). Our results confirm that filter strips saturated in whole blood stored at room temperature showed poorer performance, possibly due to protein denaturation, desiccation and pH changes that compromise antibody stability (Wang *et al.,* 2007; Kaur, 2021). Additionally, microbial growth may be encouraged at room temperature, potentially degrading antibodies and increasing false positives through non-specific binding (James and Tawfik, 2003; Bustamante-Torres *et al.,* 2021; Jiang *et al.,* 2021). Although we did not specifically test for microbial growth, no visible mould was observed during sample processing on any of the samples. Threshold optimization, as seen with the Youden index-optimized threshold of 0.8274 for filter strips stored at room temperature, improved diagnostic performance. However, these thresholds require ongoing validation to ensure stability in long-term surveillance programmes and to monitor potential increases in false positives over time.

The Nobuto filter strips used in this study hold a defined, relatively small volume of blood (approximately 0.1 ml), which can limit the number of follow-up tests that can be performed. While a fully saturated strip provided sufficient sample to conduct the IDEXX NP and NCFAD NP, H5 and H7 assays, there was often insufficient remaining sample to perform hemagglutination inhibition testing at NCFAD, which provides additional serological insights. This is particularly relevant because the H5 cELISA cannot differentiate between antibodies against highly pathogenic and low pathogenic avian influenza H5 viruses. Fully saturated strips are preferred not only to maximize sample availability but also to ensure a predictable volume of whole blood and, consequently, a consistent dilution following elution. However, achieving full saturation of the strip can be challenging in two key use cases: (1) small-bodied birds (e.g. songbirds), where collecting 0.1 ml of blood is either not advisable or practically challenging (Fig. 1c), and (2) hunter-harvested samples (e.g. heart blood), where user inexperience and/or carcass condition may result in incomplete saturation (Fig. 1c). To address these challenges, standardized protocols for adjusting elution volumes based on the degree of saturation are recommended to improve performance and interpretability in these contexts.

Species-level data are presented to facilitate comparison of quantitative values; however, establishing species-specific diagnostic thresholds or exploring interspecies performance differences was not an objective of this study. The assays used here are generally not validated across multiple species, and there is no evidence to suggest that the difference in performance between serum and whole blood is species dependent rather than a function of the sampling method itself. Instead, our focus was on identifying a broadly applicable threshold for serosurveillance in wild birds in Canada, enabling consistent interpretation of results when a standardized methodology is employed. Species-specific patterns were difficult to discern, particularly given small sample sizes in some cases, and variability appeared to be driven primarily by individual sample-level differences rather than species-level effects. While additional species-specific follow-up studies may be warranted for those seeking to refine estimates at the species level, establishing a single optimized threshold that enables comparability of results across laboratories using the same standardized methodology satisfies many priorities and is of clear value for a national surveillance programme. Given that diagnostics are conducted at regional diagnostic nodes, defining a threshold that remains consistent when applied across different laboratories supports reliable interpretation and integration of results at a national scale.

The collection of whole blood on filter strips can provide a practical tool for surveillance programmes in remote or resource-limited settings, small-bodied species and hunter-harvest programmes. However, this approach comes with trade-offs. Agreement between results from whole blood on filter strips and serum was approximately 80% even when freezing samples and using Youden index threshold optimization, meaning that approximately one in five samples remained misclassified. Serum remains the most robust sample type for maximizing diagnostic sensitivity and specificity. However, when serum collection is not logistically or practically feasible, filter strips can still yield valuable data on anti-IAV antibodies, provided that diagnostic thresholds are optimized and logistical factors such as storage conditions and testing delays are carefully managed.

In cases where the primary goal is not estimating seroprevalence but rather determining whether any individuals in a population have detectable antibodies indicating prior exposure (e.g. assessing whether a population is immunologically naïve), filter strips used to collect whole blood and stored at room temperature may be sufficient, provided that the sample quality is adequate and a sufficient number of individuals are sampled (which should be determined a priori based on expected prevalence). The Youden index-optimized thresholds and freezing filter strips were shown to improve diagnostic performance, particularly for H5 detection, and offer practical approaches for IAV surveillance in wild birds.

## Supplementary Material

Web_Material_coaf033

## Data Availability

The data underlying this article will be shared on reasonable request to the corresponding authors.
