# Correction to: Comparison of whole blood on filter strips with serum for avian influenza virus antibody detection in wild birds

**DOI:** 10.1093/conphys/coag011

**Published:** 2026-02-18

This is a correction to: Jolene A Giacinti, Ishraq Rahman, Jordan Wight, Hannah Lewis, Liam U Taylor, Jennifer F Provencher, Robert Ronconi, Yohannes Berhane, Wanhong Xu, Dmytro Zhmendak, Sailendra N Sarma, Christopher M Sharp, Joshua T Cunningham, April Hedd, Johanna-Lisa Bosch, Gregory J Robertson, Kathryn E Hargan, Andrew S Lang, Comparison of whole blood on filter strips with serum for avian influenza virus antibody detection in wild birds, *Conservation Physiology*, Volume 13, Issue 1, 2025, coaf033, https://doi.org/10.1093/conphys/coaf033..

In the originally published version of this manuscript, Figure 1 contained an error in its caption. In the following sentence, ``five'' should have read ``three'': ``Sampling sites span various regions in Canada and represent diverse wild bird species from five taxonomic families: Anatidae, Alcidae and Laridae''.

 The sentence has now been corrected to read: ``Sampling sites span various regions in Canada and represent diverse wild bird species from three taxonomic families: Anatidae, Alcidae and Laridae''.

 In addition, Figure 2A contained errors in the axes captions. The x axis was incorrectly labelled ``Filter Strip – Room Temperature (S/N)'' and the y axis ``Filter Strip – Frozen (S/N)''. These have now been corrected to ``Filter Strip (S/N)'' for the x axis and ``Serum (S/N)'' for the y axis. 

