# Supplementary material for: Comparison of whole blood on filter strips with serum for avian influenza virus antibody detection in wild birds
Source: Conserv Physiol. 2025 Jun 9;13(1):coaf033. doi: 10.1093/conphys/coaf033 (PMC12146847; doi:10.1093/conphys/coaf033)
Supplement: Web_Material_coaf033 [file web_material_coaf033.zip › Supplemental Figures.pdf]

Supplemental

Supplemental Figure 1. Analysis of sample-to-negative (S/N) values for NP antibody detection using IDEXX AI MultiS-Screen Ab ELISA in serum and whole blood on filter strips stored at room temperature. a) Scatter plot showing the correlation between S/N values, with dashed black lines representing the original threshold value; b) Bland-Altman plot comparing S/N values, with the solid red line representing the mean difference (bias) and dashed red lines indicating the limits of agreement (mean difference ± 1.96 SD), while the black dashed line at y = 0 serves as a reference; and c) Receiver operating curve (ROC) illustrating the diagnostic performance of filter strips relative to serum, with sensitivity (blue line) and specificity (red line) plotted against different cutoff thresholds. The grey dashed vertical line marks the optimal cutoff identified by the Youden index, with horizontal dashed lines indicating the corresponding sensitivity (blue) and specificity (red) at this cutoff.

a)

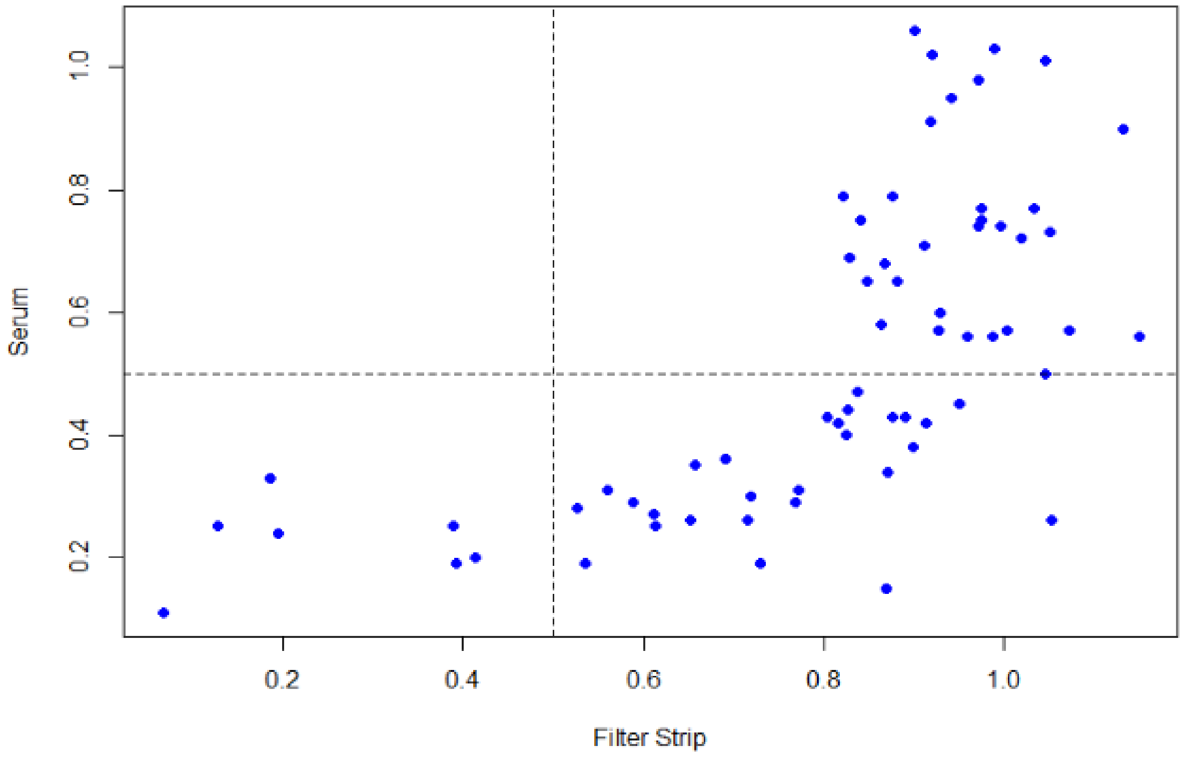

b)

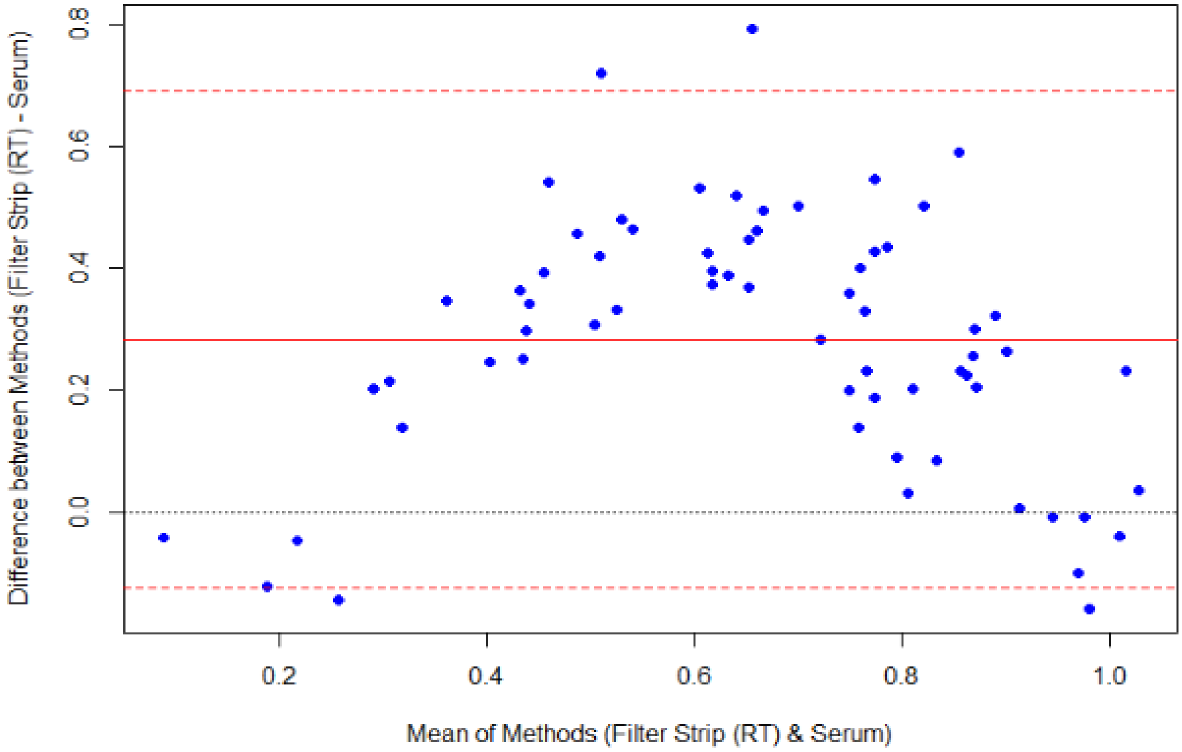
Difference between Methods (Filter Strip (RT) - Serum)
0.8
0.6
0.4
0.2
0.0
0.2
0.4
0.6
0.8
1.0
Mean of Methods (Filter Strip (RT) & Serum)

c)

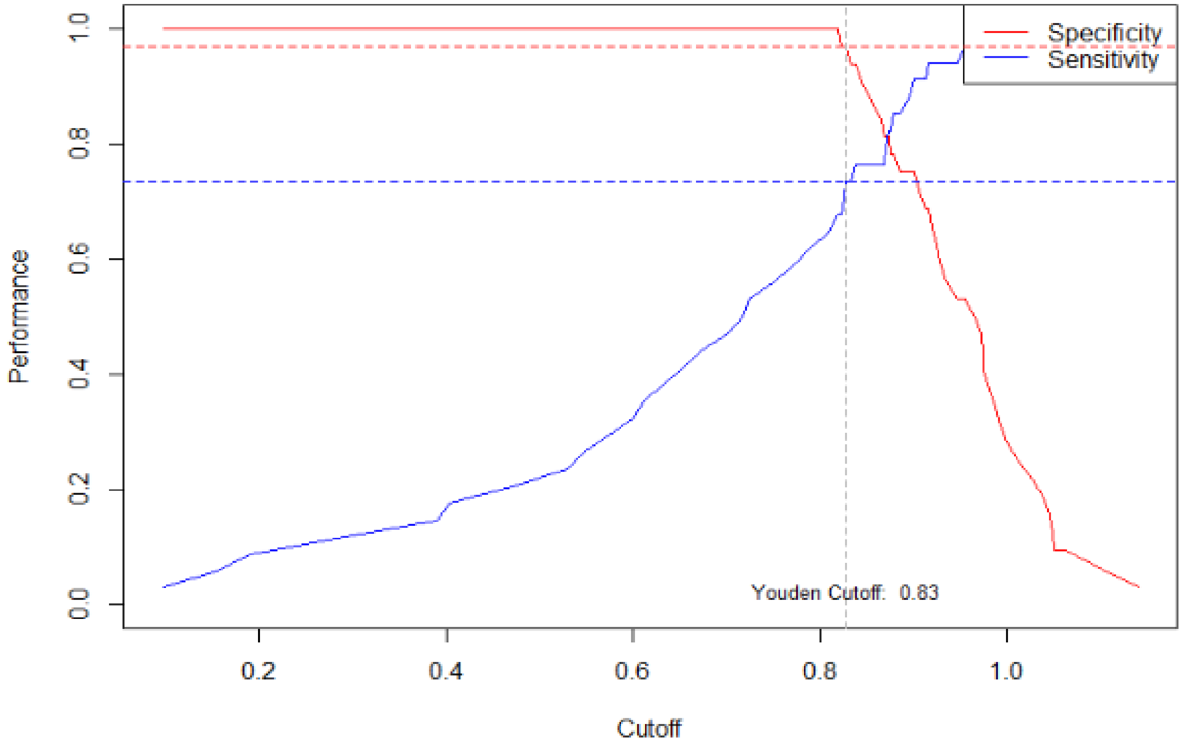
Specificity
Sensitivity
Performance
1.0
0.8
0.6
0.4
0.2
0.0
Youden Cutoff: 0.83
0.2
0.4
0.6
0.8
1.0
Cutoff

Supplemental Figure 2. Analysis of percent inhibition (PI) values for H5 antibody detection using NCFAD in house ELISA in serum and whole blood on filter strips stored at room temperature. a) Scatter plot showing the correlation between PI values, with dashed black lines representing the original threshold value; b) Bland-Altman plot comparing PI values, with the solid red line representing the mean difference (bias) and dashed red lines indicating the limits of agreement (mean difference ± 1.96 SD), while the black dashed line at y = 0 serves as a reference; and c) Receiver operating curve (ROC) illustrating the diagnostic performance of filter strips relative to serum, with sensitivity (blue line) and specificity (red line) plotted against different cutoff thresholds. The grey dashed vertical line marks the optimal cutoff identified by the Youden index, with horizontal dashed lines indicating the corresponding sensitivity (blue) and specificity (red) at this cutoff.

a)

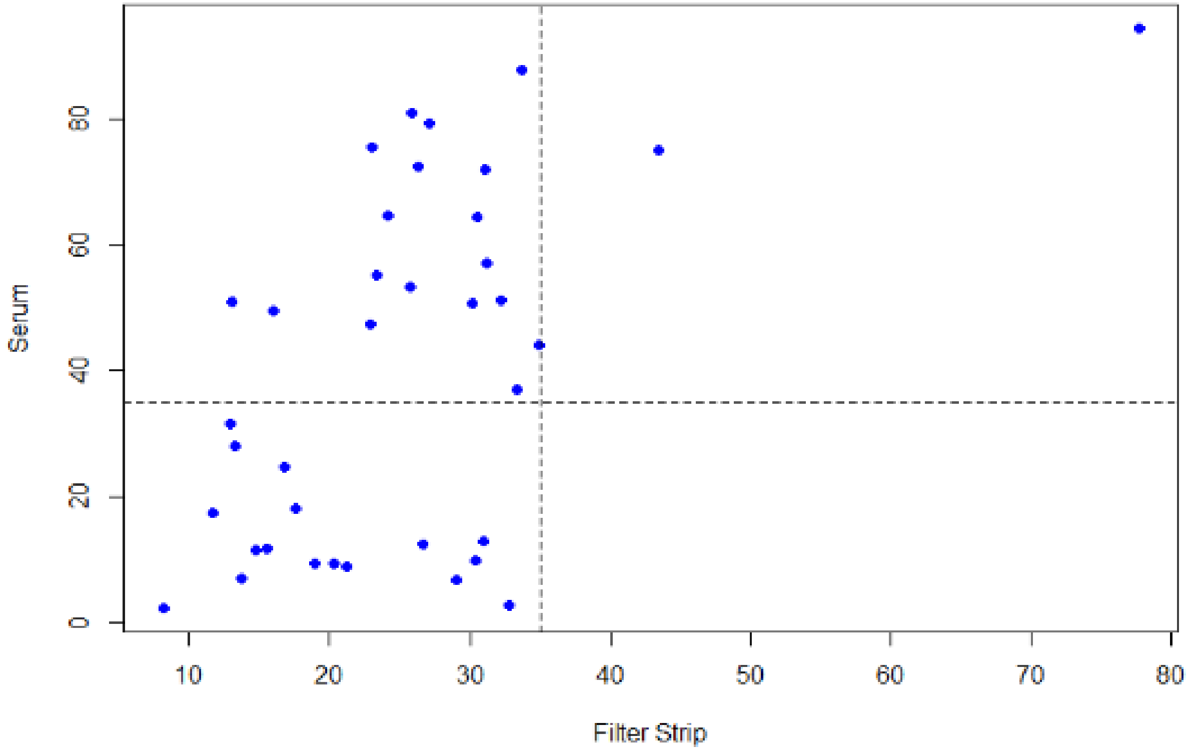

b)

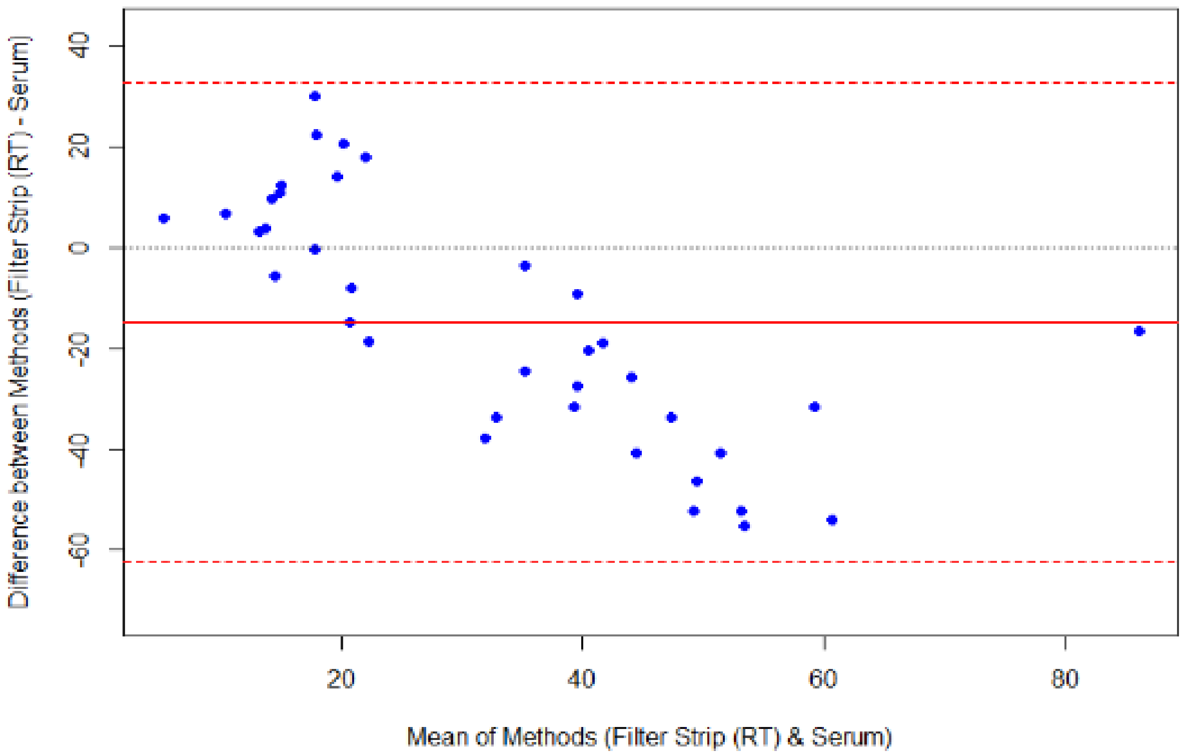
Difference between Methods (Filter Strip (RT) - Serum)
40
20
0
-20
-40
-60
20
40
60
80
Mean of Methods (Filter Strip (RT) & Serum)

c)

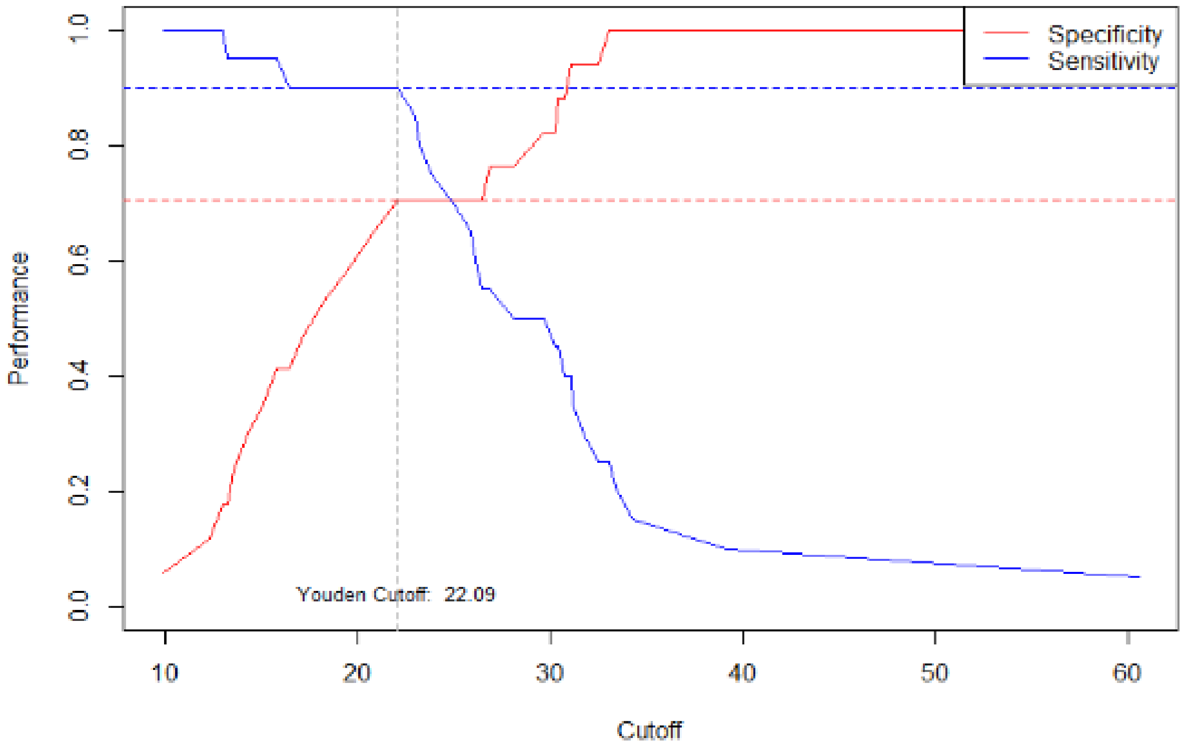
Specificity
Sensitivity
Performance
1.0
0.8
0.6
0.4
0.2
0.0
Youden Cutoff: 22.09
10
20
30
40
50
60
Cutoff

Supplemental Figure 3. Analysis of percent inhibition (PI) values for H7 antibody detection using NCFAD in house ELISA in serum and whole blood on filter strips stored frozen. a) Scatter plot showing the correlation between PI values, with dashed black lines representing the original threshold value; b) Bland-Altman plot comparing PI values, with the solid red line representing the mean difference (bias) and dashed red lines indicating the limits of agreement (mean difference ± 1.96 SD), while the black dashed line at y = 0 serves as a reference; and c) Receiver operating curve (ROC) illustrating the diagnostic performance of filter strips relative to serum, with sensitivity (blue line) and specificity (red line) plotted against different cutoff thresholds. The grey dashed vertical line marks the optimal cutoff identified by the Youden index, with horizontal dashed lines indicating the corresponding sensitivity (blue) and specificity (red) at this cutoff.

a)

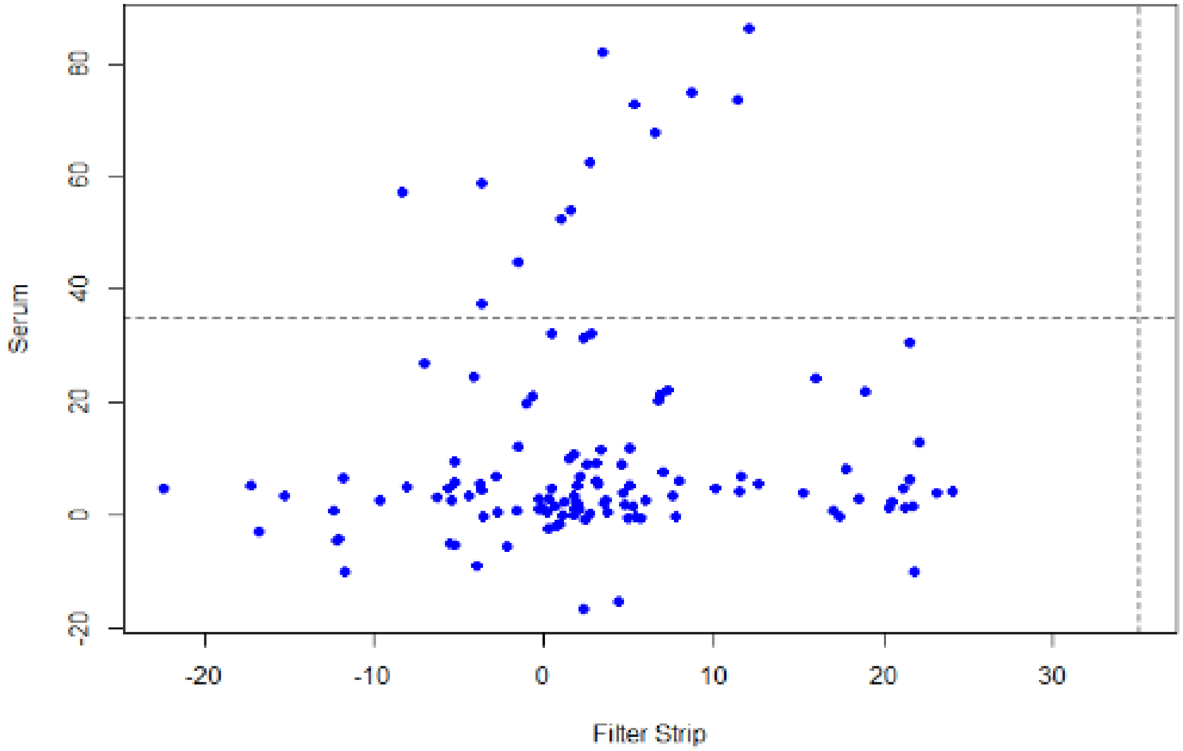

b)

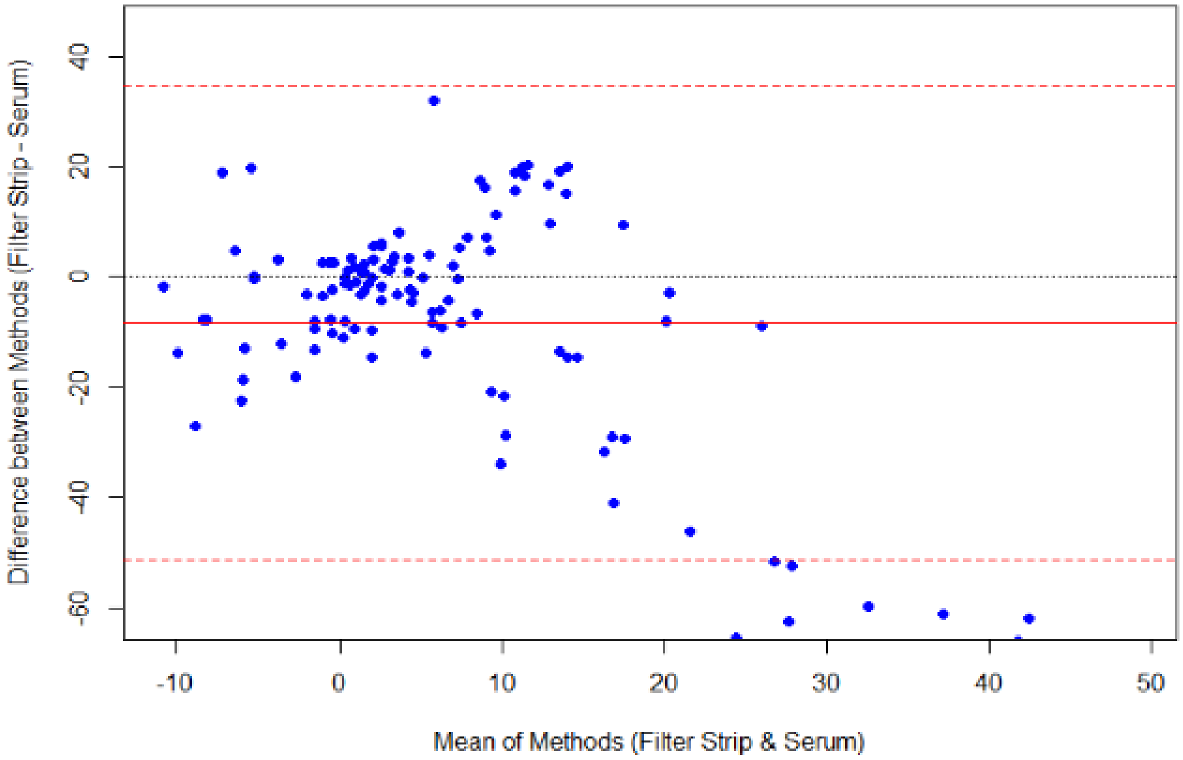

Difference between Methods (Filter Strip - Serum)
40
20
0
-20
-40
-60
-10
0
10
20
30
40
50
Mean of Methods (Filter Strip & Serum)

c)

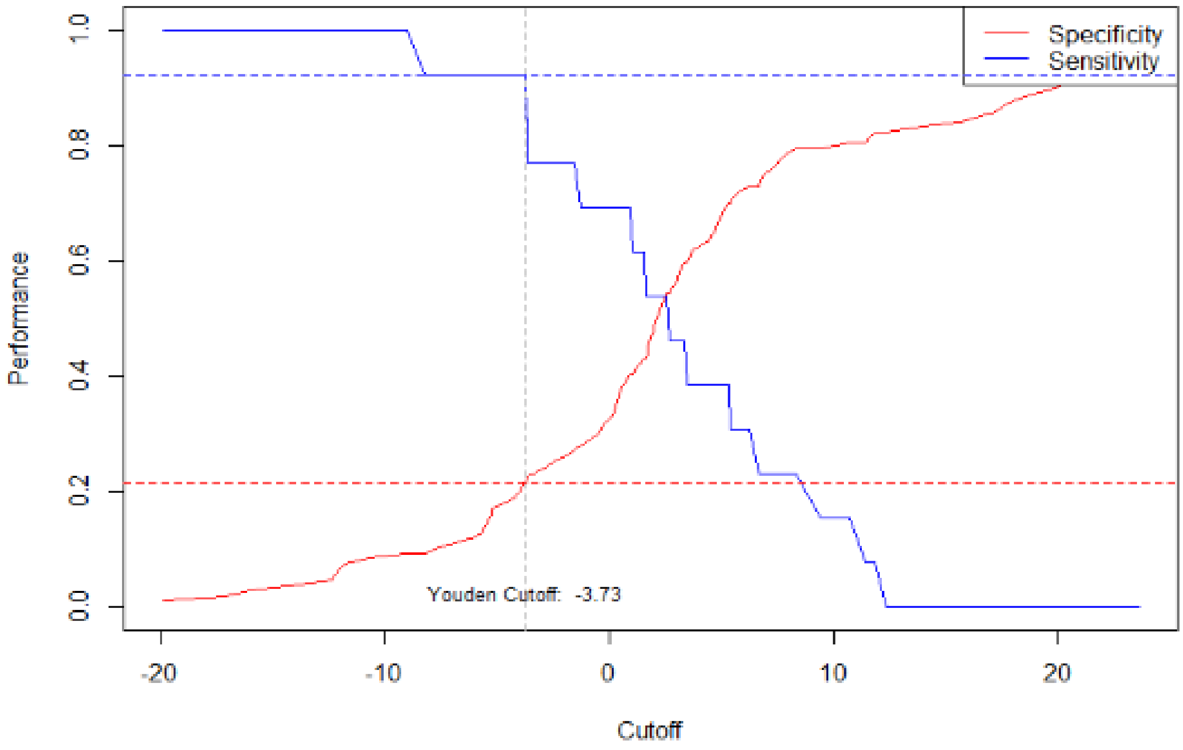

Specificity
Sensitivity
Performance
1.0
0.8
0.6
0.4
0.2
0.0
Youden Cutoff: -3.73
-20
-10
0
10
20
Cutoff
